# A Rare Case of Cerebral Amyloidoma Mimicking a Hemorrhagic Malignant Central Nervous System Neoplasm

**DOI:** 10.7759/cureus.7245

**Published:** 2020-03-11

**Authors:** Ashima Kapoor, Anusha Manje Gowda, Supreet Kaur, Elias Estifan, Michael Maroules

**Affiliations:** 1 Internal Medicine, St. Joseph's University Medical Center, Paterson, USA; 2 Hematology/Oncology, St. Joseph's University Medical Center, Paterson, USA; 3 Hematology/Oncology, St. Joseph's Regional Medical Center, Paterson, USA

**Keywords:** cerebral amyloidoma, cns lymphoma, ig a lambda plasma cells

## Abstract

Cerebral amyloidoma is a rare form of amyloidosis with a localized tumor like an amyloid deposition in the brain composed of insoluble fibrillary protein with cross beta-sheet conformation. Its usual presentation includes vision loss, seizures, behavioral changes, cognitive decline, and recurrent headaches. It has a benign course with a slow progression, and it is not associated with dissemination. We report a case of a 65-year-old Caucasian woman who presented with symptoms of progressively worsening cognitive dysfunction of six months’ duration. From CT of the brain, it was found that she had a right frontal and left parietal hemorrhagic mass with a large amount of vasogenic edema and a midline shift. MRI showed heterogeneously enhancing hemorrhagic mass of 5.2 cm x 2.6 cm x 3.6 cm in size, with a satellite lesion. Initially, this was suspected to be a high-grade glioma vs. metastatic hemorrhagic lesions. She underwent stereotactic biopsy of the mass, and histopathology was consistent with cerebral amyloidoma with marked IgA lambda plasma cell differentiation. She did not have any evidence of systemic amyloidosis, and therefore, she is being clinically observed with a regular follow-up and annual CT surveillance. She has remained stable over the past two years, although she has residual cognitive dysfunction. Cerebral amyloidoma can mimic malignant central nervous system (CNS) neoplasms and should be considered as a differential of any single or multiple mass lesions occurring in the white matter region of the brain with a characteristic appearance of “hyperdense lesions” on CT. It is a benign disease with no metastatic potential that usually resolves entirely after resection.

## Introduction

Cerebral amyloidosis is a disease that is commonly associated with aging, Alzheimer's disease, spongiform encephalitides, and cerebral amyloid angiopathy [1]. The amyloidoses are characterized by the deposition of insoluble glycoproteins in tissues. They present in two forms systemic and localized, and there is characteristic appearance on electron microscopy, apple-green birefringence in Congo red stain viewed under polarized light [1]. The most typical manifestation of central nervous system (CNS) involvement is congophilic angiopathy, in which amyloid is deposited in blood vessels, and causes cerebral hemorrhage, and also is found in neuritic plaques. Cerebral amyloidoma is the rarer form of cerebral amyloidosis with a localized tumor-like deposition of the amyloid protein.

## Case presentation

**Figure 1:**
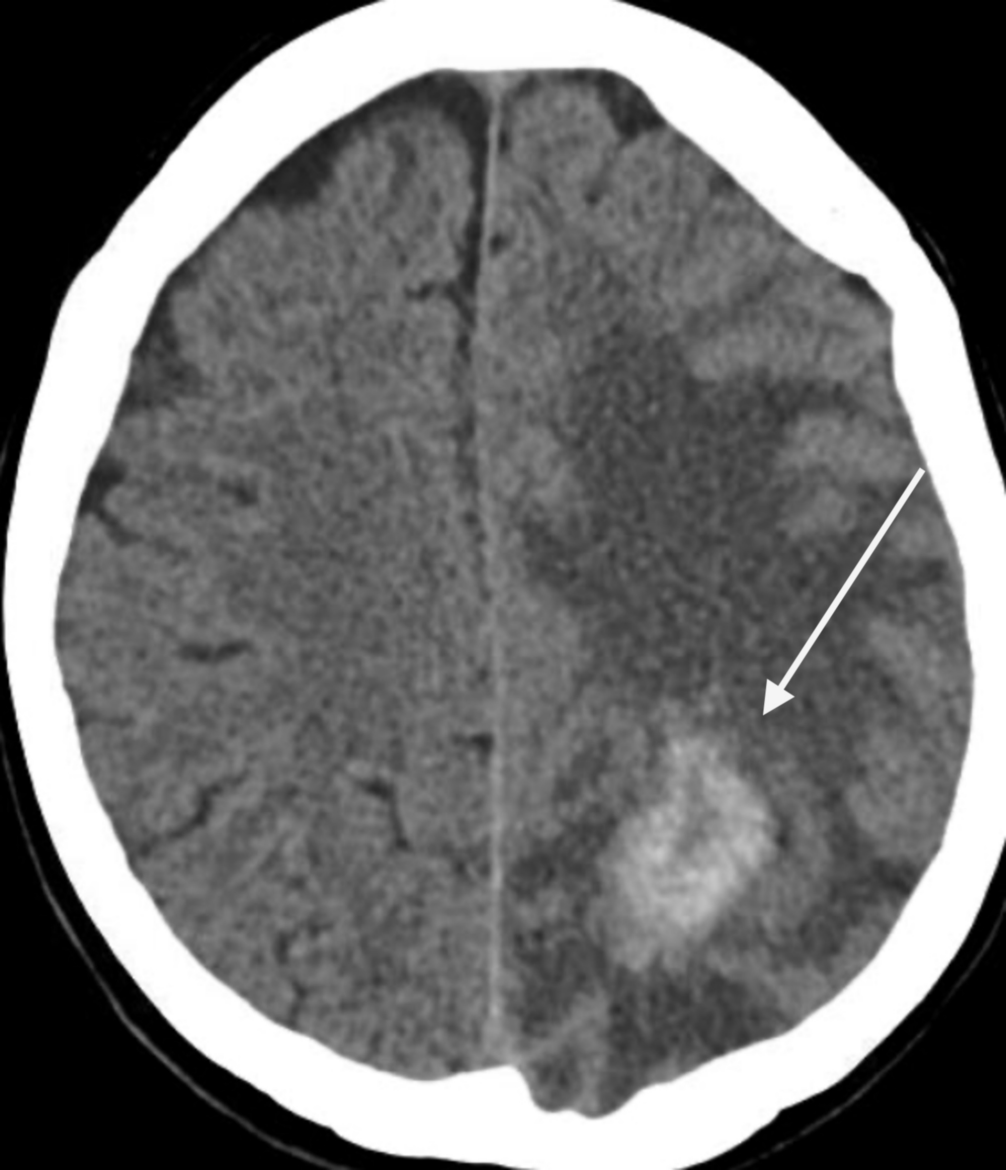
CT of the head without contrast. CT of the head without contrast showed a right-sided frontal and left-sided parietal hemorrhagic mass with a large amount of vasogenic edema associated with moderate mass effect from left to right.

**Figure 2:**
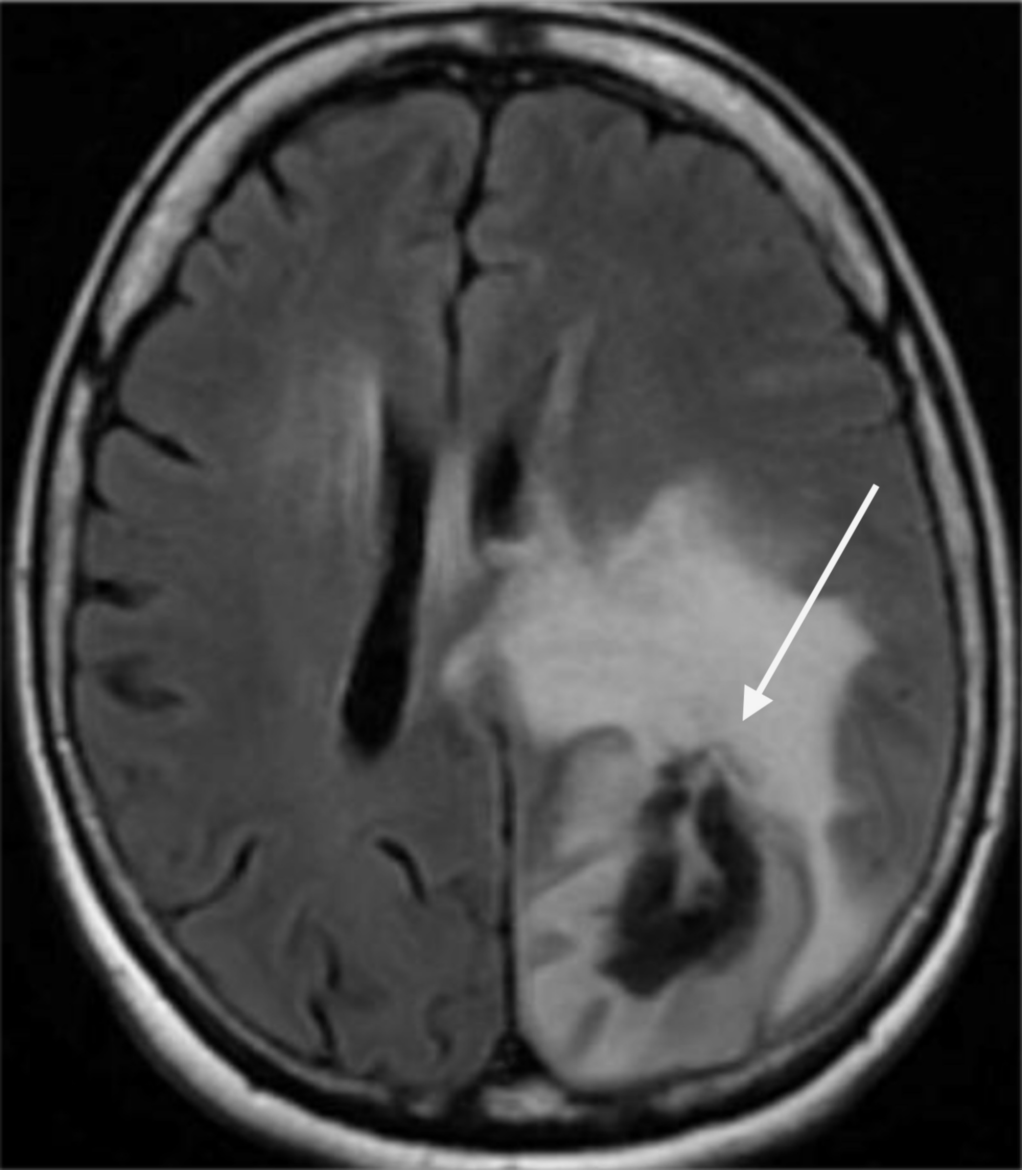
MRI of the brain. MRI of the brain showing a hemorrhagic mass within the left parieto-occipital lobe measuring 5.2 cm x 2.6 cm x 3.6 cm in size with surrounding vasogenic edema.

A 65-year-old right-handed Caucasian female with a past medical history of hypertension presented with symptoms of intermittent episodes of confusion, forgetfulness, and word-finding difficulty of six months’ duration. She had been having difficulty in completing daily tasks, remembering phone numbers, names of people, and events. She also reported intermittent frontal headaches, stabbing in nature, nonradiating, and lasting for about 20-45 min. She denied any other symptoms such as fever, weight loss, dizziness, loss of consciousness, weakness, numbness, bowel bladder incontinence, seizure activity, or gait instability. She worked as a guidance counselor for nearly 40 years and was independent in her activities of daily living. On neurological exam, she was found to have dysmetria in the right upper extremity, dysgraphia, and dysdiadochokinesia. She also had difficulty spelling words, writing sentences, and naming objects. CT of the head without contrast (Figure 1) was obtained for further evaluation. It showed a right two hemorrhagic mass lesions in the right frontal and left parietal regions with a moderate mass effect. MRI of the brain (Figure 2) was then obtained that showed a dominant heterogeneously enhancing hemorrhagic mass within the left parieto-occipital lobe measuring approximately 5.2 cm x 2.6 cm x 3.6 cm in size. An extensive surrounding vasogenic edema was also identified. The prominent mass effect upon the left lateral ventricle was seen without hydrocephalus. There was an additional satellite focus of hemorrhagic enhancing tumor within the deep white matter of the right frontal lobe measuring up to 13 mm axially.

**Figure 3:**
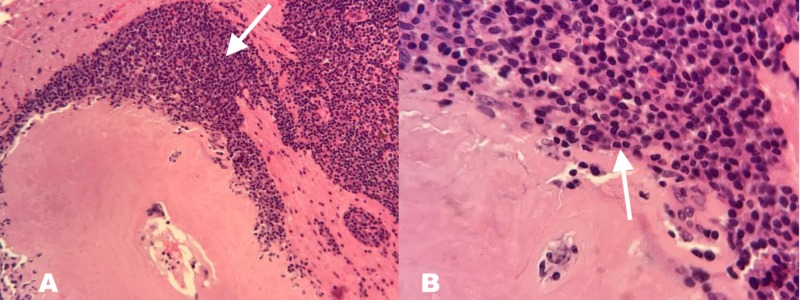
Histopathology images. A - Low-power image showing lympho-plasmacytoid infiltrate with eosinophilic material favoring cerebral amyloidoma or a lymphoplasmacytoid lymphoma (arrow). B - High-power image with H & E section showing a large areas of acellular, amorphous, and slightly eosinophilic material associated with focally dense infiltrates of small lymphocytes, plasmacytoid cells, and plasma cells. The lymphocytes lacked overt cytologic atypia (arrow), and the plasma cells were mostly mature forms.

The initial differential diagnoses were hemorrhagic metastases vs. CNS lymphoma vs. high-grade glioma of the brain. The patient was started on dexamethasone four milligrams IV every six hours along with levetiracetam 500 mg every 12 h for seizure prophylaxis. Metastatic workup, including CT of the chest, abdomen, and pelvis did not show any evidence of an occult malignancy. MRI of the brain was done with stealth protocol, and the patient underwent a left parietal burr hole biopsy of the mass. Histopathology was obtained (Figures 3) that showed a lympho-plasmacytoid infiltrate with eosinophilic material favoring cerebral amyloidoma or a lymphoplasmacytoid lymphoma. Immuno-histochemical analysis revealed a B cell population positive for CD20, PAX5 (weak), CD23, and BCL2. These cells were negative for CD5, CD10, CD43, BCL1, and MUM1. The plasma cell population (>70% of cells) was positive for CD20 (partial), CD138, MUM1, and cytoplasmic IgA lambda. Congo red stain was positive in the acellular material, consistent with cerebral amyloidoma with marked IgA lambda plasma cell differentiation.

She underwent further workup to rule out systemic amyloidosis, which was negative. She is currently being followed up in our hematology-oncology clinic, along with annual CT surveillance. Her brain lesion has remained stable over the past two years, although she has residual cognitive dysfunction.

## Discussion

Cerebral amyloidoma usually occurs as a single lesion, but multiple lesions have been reported in about one-third of the cases, and it commonly involves the supratentorial and subcortical white matter of the brain [2]. Few cases have reported amyloidoma formation at the skull base [3] within the trigeminal nerve causing trigeminal neuralgia [2-3]. Spinal and peripheral nerves may also be involved, with Gasserian ganglion being the most common site [4]. It is believed that latent infections in the ganglion might play a role as antigens that stimulate B-cells [2]. It is usually limited to a single hemisphere and may have associated satellite lesions, but one rare case of cerebral amyloidoma involving both hemispheres similar to a butterfly glioma has been reported [5]. Patients usually present with focal neurological deficits, vision loss, seizures, behavioral changes, cognitive decline, and recurrent headaches [1, 3].

Laeng et al. proposed that amyloidomas may be formed as a response to certain antigens by reactive monotypic lambda producing B-cells. Since the CNS is an immunologically protected site, there is very limited exposure to antigens. Hence cerebral amyloidoma is a rare phenomenon [6]. However, Cohen et al. postulated that genetically predisposed microglia might be stimulated to produce amyloid protein when challenged by certain antigens [7].

The histopathology of amyloidoma has been described as amyloid protein deposition with a surrounding cell infiltrate composed of monotypic kappa or lambda producing plasma cells without cytologic atypia, and few mature T lymphocytes and macrophages of the foreign body type. Apple-green birefringence under polarized light and Congo red staining is a pathognomonic feature that is universal to all forms of amyloid [6-7].

Cerebral hemorrhage can occur as a complication of cerebral amyloidoma [8-9]. It has been postulated by Labro et al. that hemorrhage occurs due to the deposition of amyloid within the brain parenchyma and cerebral vessels leading to friable vessel walls [8].

Radiological appearance: On nonenhanced CT scans, the amyloid material usually appears hyper attenuated and shows enhancement with the use of contrast but usually does not cause mass effect or perilesional edema [10]. Few cases have reported hypo attenuation on CT imaging, in the setting of contrast enhancement [11]. Amyloidomas can be hypointense, hyperintense, or isointense on T1-weighted images; the extent of amyloid protein deposition influences the signal intensity on T1-weighted images [10-11]. The signal intensity on T2-weighted images is mixed, with areas of high and low signal intensity, and are always contrast-enhancing [10-11]. Sometimes, there may be a medial extension up to the lateral ventricle ependyma with the appearance of finely irregular, radiating lines from the edge of the tumor, which may represent the deposition of amyloid along the vessels [10]. Although not routinely used, recent data have provided insight into the role of fluorodeoxyglucose-positron emission tomography (FDG-PET) which enhances the sensitivity of more than 90% and the specificity of more than 75%, when combined with clinical diagnosis [12].

Its recommended to rule out systemic amyloidosis by performing bone marrow biopsy along with serum-free light chains and immunofixation, scintigraphy, and radiological examinations, as untreated systemic amyloidosis has a poor prognosis with a mean survival time of less than one year [5, 13]. Autologous peripheral blood stem cell transplantation is the most effective therapy for systemic amyloidosis, and high dose oral melphalan with dexamethasone is recommended for patients who are not stem cell transplant candidates [14]. However, medical treatment in patients with localized amyloidoma has not been shown to be very successful; however, complete tumor resection has been shown to be effective [5]. In patients with multiple foci, surgical resection is still favorable; if the tumor involves brain territories such as sensorimotor region, speech areas, the visual cortex or the brain stem, wherein surgical resection involves significant risk and loss of function, a “wait-and-see” strategy is preferred [15]. The lesions that are not resected have a tendency to grow further in size [15]. Noninvasive treatments such as steroids, radiation, or colchicine have not shown to be effective [6].

It is hypothesized that radiation may eliminate clonal plasma cells, which are responsible for amyloid deposition [16-17]. Meier et al. reported a case of a solitary cerebral amyloidoma that was treated with fractionated radiotherapy, as the lesion could not be resected due to its anatomical location. Focal radiotherapy can be used as a therapeutic option if the anatomical location of the lesion prohibits resection [16]. It is believed that radiotherapy will not cause a regression of the amyloid that has already been deposited but prevents the progression of the lesion [16].

Cerebral amyloidoma has a benign course; no known cases of dissemination or recurrence after resection has been described so far. Renard et al. described a patient with isolated brain amyloidoma without systemic amyloidosis who was followed up for a long term, for about 17 years since diagnosis, which is the longest duration of long-term follow-up reported. The patient remained clinically stable. However, a progression in the size of the lesion was noted [18]. It has the potential for further progression in size if left unresected [5, 19] but usually does not recur after complete resection [20].

## Conclusions

We report a case of a 65-year-old Caucasian woman who was diagnosed with cerebral amyloidoma without evidence of systemic amyloidosis that presented with a hemorrhagic mass associated with significant mass effect and multiple foci that is unusual for cerebral amyloidoma. Most cases reported have described lesions without significant edema and mass effect. Cerebral amyloidoma can mimic malignant CNS neoplasms and should be considered in the differential of any single or multiple mass lesions occurring in white matter regions with a characteristic CT appearance of “hyperdense lesions” with contrast enhancement. It is usually a benign disease and slow-growing in nature with no metastatic potential. It has a very good cure rate after resection, with no reported cases of recurrence so far.
